# Statins for aortic valve stenosis

**DOI:** 10.1590/1516-3180.20161346T1

**Published:** 2016-06-03

**Authors:** Luciana Thiago, Selma Rumiko Tsuji, Jonathan Nyong, Maria Eduarda dos Santos Puga, Aécio Flávio Teixeira de Góis, Cristiane Rufino Macedo, Orsine Valente, Álvaro Nagib Atallah

## Abstract

**BACKGROUND:**

Aortic valve stenosis is the most common type of valvular heart disease in the USA and Europe. Aortic valve stenosis is considered similar to atherosclerotic disease. Some studies have evaluated statins for aortic valve stenosis.

**OBJECTIVES:**

To evaluate the effectiveness and safety of statins in aortic valve stenosis.

**METHODS:**

*Search methods:* We searched the Cochrane Central Register of Controlled Trials (CENTRAL), MEDLINE, Embase, LILACS - IBECS, Web of Science and CINAHL Plus. These databases were searched from their inception to 24 November 2015. We also searched trials in registers for ongoing trials. We used no language restrictions.

*Selection criteria:* Randomized controlled clinical trials (RCTs) comparing statins alone or in association with other systemic drugs to reduce cholesterol levels versus placebo or usual care.

*Data collection and analysis:* Primary outcomes were severity of aortic valve stenosis (evaluated by echocardiographic criteria: mean pressure gradient, valve area and aortic jet velocity), freedom from valve replacement and death from cardiovascular cause. Secondary outcomes were hospitalization for any reason, overall mortality, adverse events and patient quality of life.

Two review authors independently selected trials for inclusion, extracted data and assessed the risk of bias. The GRADE methodology was employed to assess the quality of result findings and the GRADE profiler (GRADEPRO) was used to import data from Review Manager 5.3 to create a 'Summary of findings' table.

**MAIN RESULTS:**

We included four RCTs with 2360 participants comparing statins (1185 participants) with placebo (1175 participants). We found low-quality evidence for our primary outcome of severity of aortic valve stenosis, evaluated by mean pressure gradient (mean difference (MD) -0.54, 95% confidence interval (CI) -1.88 to 0.80; participants = 1935; studies = 2), valve area (MD -0.07, 95% CI -0.28 to 0.14; participants = 127; studies = 2), and aortic jet velocity (MD -0.06, 95% CI -0.26 to 0.14; participants = 155; study = 1). Moderate-quality evidence showed no effect on freedom from valve replacement with statins (risk ratio (RR) 0.93, 95% CI 0.81 to 1.06; participants = 2360; studies = 4), and no effect on muscle pain as an adverse event (RR 0.91, 95% CI 0.75 to 1.09; participants = 2204; studies = 3; moderate-quality evidence). Low- and very low-quality evidence showed uncertainty around the effect of statins on death from cardiovascular cause (RR 0.80, 95% CI 0.56 to 1.15; participants = 2297; studies = 3; low-quality evidence) and hospitalization for any reason (RR 0.84, 95% CI 0.39 to 1.84; participants = 155; study = 1; very low-quality evidence). None of the four included studies reported on overall mortality and patient quality of life.

**AUTHORS CONCLUSIONS:**

Result findings showed uncertainty surrounding the effect of statins for aortic valve stenosis. The quality of evidence from the reported outcomes ranged from moderate to very low. These results give support to European and USA guidelines (2012 and 2014, respectively) that so far there is no clinical treatment option for aortic valve stenosis.

The full text of this review (English), the abstract (English and Polish) and the plain language summary (English and Polish) are available from: http://onlinelibrary.wiley.com/doi/10.1002/14651858.CD009571.pub2/full
